# Cardiac surgery: What the future holds?

**DOI:** 10.1186/1749-8090-6-93

**Published:** 2011-07-27

**Authors:** Haralabos Parissis

**Affiliations:** 1 Consultant Cardiothoracic Surgeon, Cardiothoracic Department, Royal Victoria Hospital, Belfast, UK

## Abstract

Cardiac surgery has been scrutinized and challenged as no other specialty has. That has brought new ideas and structural frameworks but has also brought uncertainty and scepticism.

This report identifies the challenges that the specialty is facing, and suggests solutions and strategies for the future.

## Introduction

Within the last 50 years, the steps of progress in the medical field are impressive: The 5-year cancer survival rates have risen from 30% to over 60% [1]. Cardiovascular mortality between 1950 and 1990 has decreased by about the same number & HIV/AIDS has been transformed into a chronic disease [1]. Life expectancy for a 45-year-old has increased 9 years since 1950 [2].

On the other hand, in the US only, it has been estimated that the number of citizens over the age of 75 is expected to quadruple over the next 50 years [3]; furthermore, IHD is the leading cause of death and in UK accounts for 17.4% of all deaths annually. IHD accounted for approximately one in six male deaths and one in eight female deaths during 2009 [4]. So, although the predictions outline the increased future demand of Cardiothoracic surgery, the specialty has witnessed a notable decrease in applicants over the past decade. In this context, Grover and colleagues [5] reported that the "United States will face a severe shortage of cardiothoracic surgeons within 10 years if entry into the profession keeps declining.'

### The problem

There is a lack of interest amongst young trainees for the cardiothoracic specialty and since 2003 the number of recruiters in the specialty is reducing annually.

The reason for this discrepancy is multifaceted.

### Aetiology

1) Coronary Stent technology has grown larger and has displaced Coronary Artery Bypass surgery globally; In the British Isles, three PCIs are carried out per surgically revascularised case; Furthermore, Intravascular procedures continue to evolve not only with the use of intracoronary stents but also with the introduction of such a technology for the treatment of aortic pathologies and valvular heart disease.

Each year patients who undergo cardiac surgery continue to be sicker, older, and at higher risk for complications. As patients get sicker and hear about advancing technology, they are more likely to have unrealistic expectations.

2) There is a lack of strong links between innovative research and clinical practice.

Despite this eruption of ideas, the uptake of change amongst the surgical fraternity is variable. Undoubtfully, there is a skepticism amongst the surgical community of accepting new ideas and implement them, especially when the data to support new concepts are low level of evidence, due to lack of randomize control trials. On the other hand there is the claim, that in order to manage the uncertainties of innovation one should implement surgery in a more scientific way, by drawing on the ideas of control, rationality, objectivity, and predictability [6].

3) Within the specialty they would be examples of practitioners focusing on personal development at the expense of training, or on the other hand Personal development and training slows down or even arrests after completion of training and lastly sometimes there is a reluctance of Senior practitioners to learn new techniques but that should not be allowed to block innovative practice.

4) There is a growing separation of cardiac surgery from the diagnostic process; therefore the algorithms of the treatment of three vessel disease are not well embraced by the practitioners treating those patients, resulting for example, 30% more ad hoc PCI to be carried out without robust indications [7].

So, how should cardiac surgery deal with the fact that technology changes rapidly, and that the potential therapeutic options for patients increase faster than prospective trials can evaluate them?

### Suggested solutions

Academic surgery is based on written evidence derived from prospective trials, meta-analyses, or published guidelines, edited by medical societies and this has been the cornerstones of the evolution of Cardiac surgery. However there is a need of pursuing academism in a more dynamic fashion; this should be matched with a need for a change in our professional behaviour, from the individual to the team approach of care. In fact, the entire culture of academic medicine is moving away from individualism. The tertiary care world is shifting from the achievements of individual experts toward cooperation between individuals and groups.

I urge for an attitude model of altruism and leadership: altruistic leadership based on the motivation behind helping. I suggest a model "back to the real virtues" of a commitment to leadership, scholarship, mentoring, and quality patient care.

We are surrounded by talented, highly committed individuals who want to be part of a successful team; however we need to move towards an attitude where group trust is as strong force as individual trust.

### How?

A multifaceted approach has to be adapted, by improving cardiothoracic surgical resident education, developing innovative techniques for both resident and postgraduate education (including diversification of key clinical skill sets to include catheter-based training and simulation/electronic learning as learning tools), and redesigning the current resident training paradigm.

The development of an educational body with a specific task to broaden the appeal of cardiothoracic training to new recruits, should be considered. The duty of such a body should be focus in investigating avenues of increased exposure for the cardiothoracic specialty, including a greater internet presence not only on professional society Web sites but also on contemporary social Web sites, such as "Facebook" or direct marketing the High schools. This should be an effort on attracting "high-calibre individuals or "The Best and the Brightest" as per Kim et al [8].

Finally, there is evidence that "established, mature" cardiothoracic surgeons can play a very powerful role simply through their interactions with students at any level.

There is also clear evidence [9] that academic mentoring of medical students in their early formative years has a profound effect on guiding them into surgery as a career choice.

What is the future of Cardiac Surgery?

Although, the answer brings to mind futuristic technology, I think that the future needs to be laid with care, by taking on board the lessons of the past; the innovation has to be bridged to the clinical practise. This could be achieved by linking the basic research to its clinical application (translational medical research) by ensuring for example, that Academic leaders take up new roles in the health service. The result is that world-class researchers work alongside their clinical counterparts to ensure that research and education inform and are informed by, clinical need. [10]. The Academic Health Science Centre model, which is a partnership between a healthcare provider and a University, may be the way forward.

### Predictions for the future

There is a suspicion on implementing new technology: McKinlay [11] argued that many, if not most, innovations in medicine undergo a process of which assessment of effectiveness is only a late stage placing many patients at risk of receiving treatments which are useless or malign.

So, how does a society decide which new medicines, technologies and tests should become available to all of its citizens? How do these national decisions fit into and affect a global pattern of healthcare delivery?

I would propose that only education and scientific backup would allow authorities to overcome hesitancy on taking up new ideas and innovative practices. Educating health members, aids to eliminate the gap between practitioners: For the older to become familiar with the new technology and for the younger to learn from previous experience.

Finally, a word of caution: Currently, significant and often unperceived conflicts of interest exist for everyone involved in delivering health care, and hence it is difficult for the patient to make a well- informed opinion. The answer to this problem may be the formation of "Interdisciplinary working groups" in order to facilitate robust informed consents and patients education.

The specialty of cardiac surgery has come a long way, and now it stands between crossroads. The future is the new recruiters, the young learners; in order to get the best out of them, teachers must teach differently. New technology and skill sets are necessary for thoracic surgery to grow and flourish. We must change even though change is not easy.

We are on the threshold of a brave new world in which the measurement of surgical performance will no longer be peripheral to our work, but an integral part of it.

## Conclusion

Innovation studies probably fallen out of "fashion" with an interest in new technologies and how they were validated; the fear on implementing innovational new ideas should be tackled with educational, critical mind. Robust level of evidence derives from multi-centre prospective randomized trials. Authorities and medical practitioners should be working towards implementing those principles.
